# Machine learning predicts significant improvement in motor aphasia with tongue acupuncture

**DOI:** 10.3389/fneur.2025.1554208

**Published:** 2025-10-01

**Authors:** Qixiu Wang, Guoce Zhan, Hongfei Zhou, Jun Liu

**Affiliations:** ^1^Liaoning University of Traditional Chinese Medicine, Liaoning, China; ^2^Affiliated Hospital of Liaoning University of Traditional Chinese Medicine, Liaoning, China

**Keywords:** motor aphasia, tongue acupuncture, clinical characteristics, machine learning, propensity score matching

## Abstract

**Objective:**

Motor aphasia is a common language disorder that significantly disrupts patients’ communication abilities and quality of life. Recent studies have shown that acupuncture treatment is effective for motor aphasia, but in clinical practice, the selection of acupuncture points for motor aphasia is diverse and lacks a unified standard. Therefore, by analyzing a range of clinical parameters encompassing multiple acupuncture points, we identified independent predictive factors for recovery from motor aphasia following acupuncture treatment.

**Materials and methods:**

This retrospective case–control study included 144 patients with motor aphasia at Liaoning University of Traditional Chinese Medicine Affiliated Hospital (2019–2023). Propensity score matching (PSM) balanced baseline characteristics (age, gender, disease factors, comorbidities) using 1:1 nearest neighbor matching (caliper = 0.2 SD). LASSO, Random Survival Forest, and Gradient Boosting Machine algorithms selected 44 variables, and a multivariate Cox regression model assessed treatment outcomes.

**Results:**

After PSM, baseline characteristics were balanced between the treatment group (tongue acupuncture, *n* = 40) and the control group (*n* = 40) (SMD < 0.1). Cross-analysis using LASSO, RSF, and GBM confirmed that age, time to rehabilitation start (TSR), and tongue acupuncture treatment are key predictive factors. Multivariate Cox regression analysis revealed that age ≥60 years (HR = 0.10, 95% CI: 0.02–0.50, *p* = 0.005) and TSR ≥ 12 days (HR = 0.41, 95% CI: 0.20–0.82, *p* = 0.031) are risk factors for recovery, while tongue acupuncture treatment (HR = 2.92, 95% CI: 1.29–6.62, *p* = 0.010) significantly improved treatment outcomes. Model performance was robust, with AUC values of 0.91 ± 0.07, 0.89 ± 0.08, and 0.89 ± 0.07 for LASSO, RSF, and GBM, respectively, and Cox model AUC of 0.88. Patients were categorized into low-risk (age <60 years, TSR < 12 days, receiving tongue acupuncture) and high-risk groups, with significant differences observed (HR = 0.31, 95% CI: 0.16–0.61, *p* < 0.001).

**Conclusion:**

Tongue acupuncture enhances motor aphasia recovery, while older age and delayed rehabilitation hinder it. PSM and machine learning ensured robust predictions, supporting early tongue acupuncture. Future multicenter studies will further validate these findings.

## 1 Introduction

Motor aphasia refers to a language disorder characterized by chronic nonfluent speech, commonly seen in the course of diseases such as stroke, brain tumors, and brain trauma (1, 2). Aphasia is not only associated with more severe conditions and higher mortality rates but also significantly disrupts patients’ communication abilities and quality of life for a long time (3, 4). A large-scale study surveyed the quality of life scores of 66,000 long-term care residents and reported the strongest negative correlation between individual quality of life scores and aphasia (5). Currently, treatment measures for motor aphasia typically include etiological treatment, noninvasive brain stimulation, and speech rehabilitation, which include speech and language therapy (SLT) (6), constraint-induced language therapy (CILT) (7), and basic form processing (TUF) (8), among other behavioral treatment programs. However, the efficacy of behavioral treatment programs shows significant variability. For example, several large randomized controlled trials on SLT for aphasia have provided evidence that patients with severe aphasia respond poorly to treatment (9, 10). Therefore, discovering potential treatment strategies is crucial for improving motor aphasia.

Acupuncture therapy is an important component of complementary and alternative medicine. Over the past few decades, many studies have reported the clinical efficacy and potential mechanisms of acupuncture in treating brain diseases (11, 12). Currently, acupuncture is recommended as a complementary and alternative therapy for poststroke aphasia (13). In recent years, several meta-analyses and randomized controlled studies have shown that acupuncture can significantly improve motor aphasia after stroke (13–17). Neuroimaging studies have shown that the mechanism of acupuncture may be related to the activation and functional connection of language-related brain regions, such as the left inferior temporal gyrus, superior temporal gyrus, middle frontal gyrus, and the areas around Broca’s and Wernicke’s areas, indicating the benefits of brain functional reorganization after acupuncture (12). However, in clinical practice, the selection of acupuncture points for motor aphasia is diverse and lacks a unified standard. Therefore, identifying effective acupuncture treatment points is very important because it not only is related to the treatment efficacy but also more effectively reduces patient trauma.

To solve this problem, different methods can be used. The Cox proportional hazards model is capable of determining which clinical factors have a substantial impact on patient outcomes, but it usually requires linear patient data (18). The least absolute shrinkage and selection operator (LASSO) analysis method can construct a penalty function by shrinking regression coefficients and setting some of them to zero, resulting in a more refined model. It can handle a large number of potential predictive variables and select the most relevant variables for the disease (19). The random survival forest (RSF) model is one of the most widely used machine learning methods; it is capable of detecting relationships in complex datasets and can be used to analyze survival data (20). Furthermore, it diminishes variability and inaccuracy by incorporating the entire set of gathered data and systematically evaluating non-linear relationships and intricate interplays (21). The gradient boosting machine (GBM) is a type of boosting algorithm with higher predictive performance than random forests and has the effect of preventing overfitting (22). Therefore, in this study, we proposed three models, namely, the Lasso, RSF, and GBM models, which are based on machine learning, as well as the Cox regression model, to identify survival predictors for motor aphasia patients by integrating clinical characteristics and acupuncture sites to assess treatment outcomes, especially by identifying effective acupuncture treatment points.

## 2 Materials and methods

### 2.1 Patients and treatment

**Figure 1:**
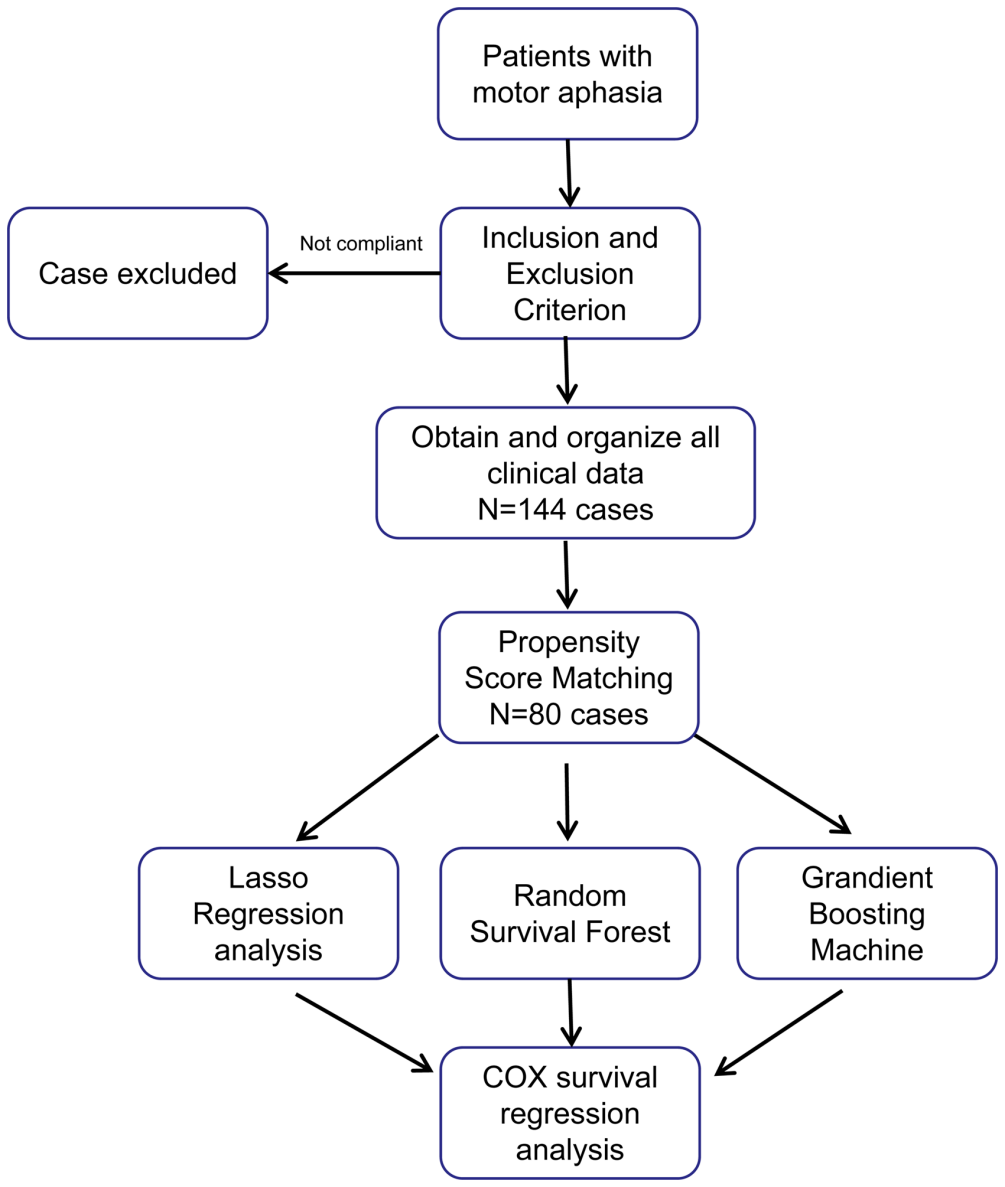
The process of this study.

This was a retrospective case–control study. Patients who were diagnosed with motor aphasia at the Affiliated Hospital of Liaoning University of Traditional Chinese Medicine between January 2019 and June 2023 were consecutively enrolled. All patients received etiological treatment according to disease guidelines and were transferred to our department. (1) Inclusion criteria: Age 18–80 years, post-stroke motor aphasia, onset within 6 months, and clear consciousness. (2) Exclusion criteria: Varying degrees of coma, history of aphasia, limb paralysis, psychiatric disorders; consciousness impairment (Glasgow Coma Scale, GCS < 12); significant cardiac, hepatic, or renal dysfunction, or other complications affecting rehabilitation; inability to complete the WAB assessment or refusal to participate in the study. All patients received rehabilitation treatment, including acupuncture and speech behavior therapy. The project was approved by the Independent Ethics Committee of the Affiliated Hospital of Liaoning University of Traditional Chinese Medicine, and informed consent was obtained from all patients. The research process is shown in Figure 1.

### 2.2 Data collection and definition

This study collected 44 indicators, including demographic and clinical characteristics and clinical imaging data. All continuous variables were converted into categorical variables based on the receiver operating characteristic curve threshold point. The collected clinical characteristics of the patients included age, sex, primary disease, motor aphasia central damage, coexisting underlying diseases, time to start rehabilitation, duration of rehabilitation, and Western Aphasia Battery (WAB) (23). The acupuncture points included Baihui (GV20), Sishencong (EX-HN1), Yamen (GV15), Fengchi (GB20), Fengfu (GV16), Yifeng (TE17), Shenting (GV24), Lianquan (CV23), Shuigou (GV26), Tongli (HT5), Neiguan (PC6), Hegu (LI4), Zusanli (ST36), Sanyinjiao (SP6), Taixi (KI3), Tai Chong (LR3), Jinjin (EX-HN12), Yu Ye (EX-HN13), language zone I, language zone II, language zone III, tongue-acupuncture points, and 30 other acupuncture points. All treatments adhered to the complete acupuncture protocol outlined in the Standards for Reporting Interventions in Clinical Trials of Acupuncture (STRICTA) guidelines (24, 25). Supplementary Figure S1 illustrates the anatomical locations of the tongue acupuncture points and related sites. Details of the tongue acupuncture technique parameters and the standardized language therapy protocol can be found in Supplementary Table S1. Motor aphasia-related central damage and the degree of brain atrophy were determined based on T2-weighted MR imaging of the brain.

We used the WAB as the evaluation standard for patients with expressive aphasia before and after rehabilitation. The WAB is a standardized and systematic assessment tool widely utilized for diagnosing and evaluating the type, severity, and related language impairments of adult aphasia. It comprehensively assesses patients’ language and cognitive functions through multiple tests, including Spontaneous Speech, Auditory Comprehension, Repetition, Naming, and Reading and Writing. The WAB generates a composite score ranging from 0 to 100, quantifying the overall severity of language function. In this study, the change in score was used to reflect the effectiveness of rehabilitation. The median change in scores was calculated, with values above the median defined as significant efficacy and those below as non-significant. Follow-up time was measured from the start of rehabilitation upon transfer to our department until discharge.

### 2.3 Propensity score matching

This study employed PSM to control for selection bias and confounding factors (26). PSM estimates the conditional probability of treatment for each subject (i.e., the propensity score) to match individuals with similar characteristics between the treatment group and the control group, thereby constructing a comparable control group (27, 28). The treatment variable is Tongue Acupuncture. Matching variables include demographic characteristics (age, sex), disease-related factors (TSR, primary disease, injury area), and comorbid conditions (hypertension, diabetes, hydrocephalus, brain atrophy, Parkinson’s disease, dementia, depressive disorders, anxiety disorders). A 1:1 nearest neighbor matching method was utilized with a caliper set at 0.2 standard deviations, employing non-replacement sampling. To ensure matching quality, continuous variables were standardized, and categorical variables were converted to dummy variables. The balance of covariates before and after matching was assessed using Standardized Mean Difference (SMD), with SMD < 0.1 typically indicating good balance (29, 30). Propensity score estimation was performed using the “scikit-learn” library, with a custom function implemented for nearest neighbor matching.

### 2.4 RSF model

The optimal cut-off values for continuous variables were determined using a custom Python script based on the maximum statistic method and converted into categorical variables. Enrolled patients were randomly divided into training and test groups (7:3 ratio) using random sampling to generate the training dataset. Random survival forest analysis was conducted using the RandomSurvivalForest class from the Python package scikit-survival (version 0.20.0). During training, multiple random decision trees were generated to form the random survival forest. Each decision tree contributed a prediction, and the final model prediction was determined by majority voting across all trees. Feature selection was based on out-of-bag (OOB) error, with the Gini index used to select splitting attributes. Variable importance was assessed using the feature_importances_ attribute, where higher values of mean decrease in accuracy and mean decrease in Gini indicate greater variable importance. To optimize model performance, five-fold cross-validation was performed using sklearn.model_selection. K Fold to evaluate the model’s robustness across different data splits, ensuring stable feature selection and prediction accuracy.

### 2.5 LASSO regression analysis

LASSO regression analysis was performed using the Lasso class from the Python package scikit-learn (version 1.3.0). LASSO regression is suitable for large datasets with numerous variables. The regularization parameter alpha controls the strength of the penalty; a larger alpha results in greater penalization, fewer retained variables, and lower model complexity. The optimal alpha was selected via five-fold cross-validation using the LassoCV class, and variables with coefficients of zero were excluded. After model construction, performance was evaluated on the test set, with results visualized using the receiver operating characteristic (ROC) curve (via sklearn.metrics.roc_curve) and confusion matrix (via sklearn.metrics.confusion_matrix). Five-fold cross-validation was applied to assess model stability and generalization, ensuring robust variable selection and predictive performance.

### 2.6 GBM model

Gradient boosting machine analysis was conducted using the XGBClassifier or XGBRegressor (depending on the survival analysis task) from the Python package xgboost (version 2.0.0), integrated with scikit-survival for survival data handling. The model was trained on the training data with 1,000 iterations. The trained model was used to predict outcomes on the test set, and performance was evaluated by calculating the mean squared error (MSE, via sklearn.metrics.mean_squared_error) and constructing a confusion matrix (via sklearn.metrics.confusion_matrix), visualized using matplotlib or seaborn. Feature contributions to model predictions were obtained via the feature_importances_ attribute and visualized. Five-fold cross-validation was implemented using sklearn.model_selection. KFold to optimize hyperparameters and evaluate model generalization, ensuring robust performance across data splits.

### 2.7 COX regression model

The Cox regression model was constructed using the CoxPHFitter class from the Python package lifelines (version 0.27.7) to build both univariate and multivariate models, evaluating factors associated with survival and generating hazard ratios (HR) with confidence intervals (CI). Based on the model results, patients were divided into high-risk and low-risk groups. Kaplan–Meier survival curves for these groups were generated using the KaplanMeierFitter class and visualized with matplotlib. The proportional hazards assumption was tested using the check_assumptions method in lifelines to ensure model validity. Five-fold cross-validation was performed using sklearn.model_selection. KFold to assess the stability of the Cox model’s coefficients and predictive performance across different data folds.

### 2.8 Statistical analysis

We used Python software V3.10.8 for statistical description and analysis. Independent sample t tests and chi-square tests were used to investigate the differences in clinical characteristics between patients with different treatment outcomes. All tests were two-sided, and a 95% CI was used. A *p* value of <0.05 was considered to indicate statistical significance.

## 3 Results

### 3.1 Patient data characteristics and univariate analysis

A total of 144 patients with motor aphasia were included in this study. Specific data can be found in Supplementary Table S2. Patients were divided into two groups according to the median difference in the rehabilitation score before and after recovery: significant improvement (72 patients) and nonsignificant improvement (72 patients). We performed univariate analysis (two-sample *t* tests and chi-square tests) on 43 indicators of motor aphasia patients, including demographic characteristics, clinical characteristics, imaging characteristics, and acupuncture points, and the results showed that there were statistically significant differences in age, time to start rehabilitation (TSR), degree of brain atrophy, tongue-acupuncture, and rehabilitation time between the two groups of patients, while other data did not reach statistical significance (Table 1). Although 20.8% of patients in the “non-significant improvement” group received tongue acupuncture, this proportion was significantly lower than the 81.9% in the “significant improvement” group, indicating an association between tongue acupuncture and treatment efficacy (*p* < 0.001). Supplementary Table S2 indicates that the WAB-AQ scores in the tongue acupuncture group were significantly higher than those in the control group after treatment.

**Table 1:** Baseline and comparison of clinical characteristics of patients with motor aphasia.

Characteristic	Significant improvement (*N* = 72)	Unsignificant improvement (*N* = 72)	*p*
Time of tongue acupuncture(days)			
Mean (SD)	26.9 (4.31)	28.0 (4.19)	0.09
Sex			0.09
Female	39 (54.2%)	29 (40.3%)	
Male	33 (45.8%)	43 (59.7%)	
Age			0.05
Mean (SD)	55.4 (11.9)	58.9 (10.7)	
Median [Min, Max]	56.0 [37.0, 83.0]	60.0 [36.0, 81.0]	
TSR*			<0.001
Mean (SD)	9.28 (2.59)	16.4 (3.91)	
Median [Min, Max]	9.00 [0, 15.0]	15.5 [11.0, 25.0]	
Primary_disease			0.17
Brain tumor	7 (9.7%)	15 (20.8%)	
Cerebral emorrhage	32 (44.4%)	30 (41.7%)	
Cerebral infarction	33 (45.8%)	27 (37.5%)	
Damaged_area			0.24
Core	33 (45.8%)	40 (55.6%)	
Other	39 (54.2%)	32 (44.4%)	
Hypertension			0.32
No	37 (51.4%)	31 (43.1%)	
Yes	35 (48.6%)	41 (56.9%)	
Diabetes			0.49
No	45 (62.5%)	41 (56.9%)	
Yes	27 (37.5%)	31 (43.1%)	
Cerebral_atrophy			0.04
No	60 (83.3%)	50 (69.4%)	
Yes	12 (16.7%)	22 (30.6%)	
Parkinsons_disease			1.00
No	69 (95.8%)	69 (95.8%)	
Yes	3 (4.2%)	3 (4.2%)	
Depression_disorder			0.31
No	40 (55.6%)	46 (63.9%)	
Yes	32 (44.4%)	26 (36.1%)	
Anxiety_disorder			0.73
No	45 (62.5%)	44 (61.1%)	
Yes	27 (37.5%)	28 (38.9%)	
GV20			0.86
Untreated	45 (62.5%)	47 (65.3%)	
Treated	27 (37.5%)	25 (34.7%)	
EX_HN1			0.86
Untreated	39 (54.2%)	38 (52.8%)	
Treated	33 (45.8%)	34 (47.2%)	
GV15			0.86
Untreated	36 (50.0%)	37 (51.4%)	
Treated	36 (50.0%)	35 (48.6%)	
GB20			0.50
Untreated	42 (58.3%)	38 (52.8%)	
Treated	30 (41.7%)	34 (47.2%)	
GV16			0.86
Untreated	29 (40.3%)	30 (41.7%)	
Treated	43 (59.7%)	42 (58.3%)	
TE17			0.24
Untreated	42 (58.3%)	35 (48.6%)	
Treated	30 (41.7%)	37 (51.4%)	
GV24			0.49
Untreated	45 (62.5%)	41 (56.9%)	
Treated	27 (37.5%)	31 (43.1%)	
CV23			0.73
Untreated	37 (51.4%)	35 (48.6%)	
Treated	35 (48.6%)	37 (51.4%)	
CV24			0.18
Untreated	32 (44.4%)	40 (55.6%)	
Treated	40 (55.6%)	32 (44.4%)	
GV26			0.86
Untreated	29 (40.3%)	28 (38.9%)	
Treated	43 (59.7%)	44 (61.1%)	
EX_HN12			0.06
Untreated	37 (51.4%)	26 (36.1%)	
Treated	35 (48.6%)	46 (63.9%)	
EX_HN13			0.31
Untreated	38 (52.8%)	32 (44.4%)	
Treated	34 (47.2%)	40 (55.6%)	
EX_HN10			1.00
Untreated	37 (51.4%)	37 (51.4%)	
Treated	35 (48.6%)	35 (48.6%)	
HT5			0.40
Untreated	39 (54.2%)	34 (47.2%)	
Treated	33 (45.8%)	38 (52.8%)	
PC6			0.40
Untreated	41 (56.9%)	36 (50.0%)	
Treated	31 (43.1%)	36 (50.0%)	0.18
LI4			
Untreated	37 (51.4%)	45 (62.5%)	
Treated	35 (48.6%)	27 (37.5%)	
ST36			1.00
Untreated	40 (55.6%)	40 (55.6%)	
Treated	32 (44.4%)	32 (44.4%)	
SP6			0.30
Untreated	40 (55.6%)	46 (63.9%)	
Treated	32 (44.4%)	26 (36.1%)	
KI3			1.00
Untreated	41 (56.9%)	41 (56.9%)	
Treated	31 (43.1%)	31 (43.1%)	
LR3			1.00
Untreated	38 (52.8%)	38 (52.8%)	
Treated	34 (47.2%)	34 (47.2%)	
Language_Zone_I			0.06
Untreated	27 (37.5%)	38 (52.8%)	
Treated	45 (62.5%)	34 (47.2%)	
Language_Zone_II			0.49
Untreated	30 (41.7%)	26 (36.1%)	
Treated	42 (58.3%)	46 (63.9%)	
Language_Zone_III			0.40
Untreated	36 (50.0%)	31 (43.1%)	
Treated	36 (50.0%)	41 (56.9%)	
Cardiac_region			0.61
Untreated	30 (41.7%)	33 (45.8%)	
Treated	42 (58.3%)	39 (54.2%)	
Hepatic_region			1.00
Untreated	30 (41.7%)	30 (41.7%)	
Treated	42 (58.3%)	42 (58.3%)	
Splenic_region			0.31
Untreated	36 (50.0%)	30 (41.7%)	
Treated	36 (50.0%)	42 (58.3%)	
Renal_region			1.00
Untreated	31 (43.1%)	31 (43.1%)	
Treated	41 (56.9%)	41 (56.9%)	
Upper_focus_area			1.00
Untreated	32 (44.4%)	32 (44.4%)	
Treated	40 (55.6%)	40 (55.6%)	
Lower_focal_area			0.09
Untreated	34 (47.2%)	44 (61.1%)	
Treated	38 (52.8%)	28 (38.9%)	
Tongue_acupuncture			<0.001
Treated	59 (81.9%)	15 (20.8%)	
Untreated	13 (18.1%)	57 (79.2%)	

*TSR, time of starting rehabilitation.

**Figure 2:**
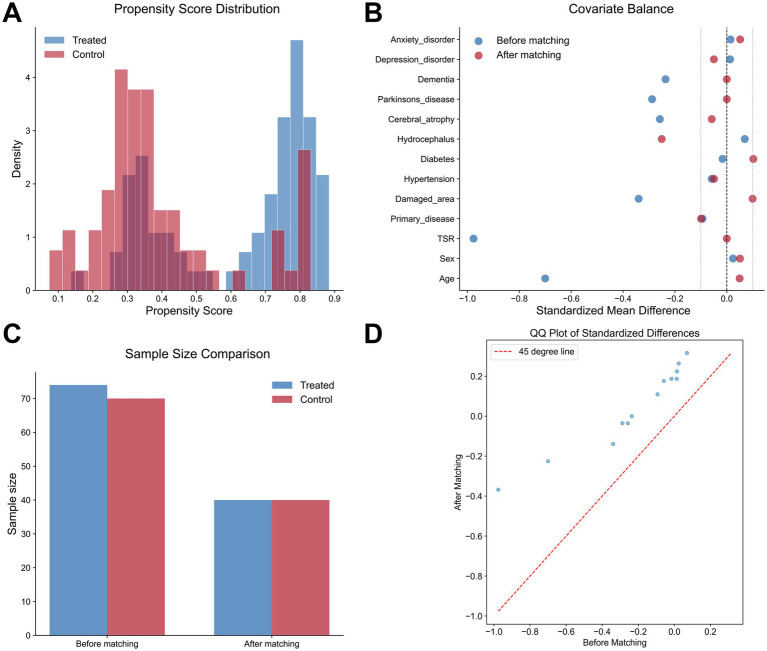
Results of Propensity Score Matching (PSM). **(A)** Propensity Score Distribution. The distribution of propensity scores for the Tongue Acupuncture treatment group (*n* = 40, solid line) and the control group (*n* = 40, dashed line) before and after 1:1 matching, exhibiting a near-normal distribution. **(B,C)** Covariate balance. **(B)** Illustrates the Standardized Mean Differences (SMD) of covariates (e.g., age, sex, WAB-AQ scores) before PSM; **(C)** shows that after PSM, most variables have SMD < 0.1, indicating balance in baseline characteristics between the two groups. **(D)** QQ plot. The QQ plot verifies the standard deviation of propensity scores before and after PSM, with *p* = 0.82 (Kolmogorov–Smirnov test), confirming the robustness of the matching process.

To address selection bias caused by potential confounding factors, this study employed PSM to balance baseline characteristics between the Tongue Acupuncture treatment group and the control group. We utilized a 1:1 nearest neighbor matching method, using Tongue Acupuncture as the treatment variable and including key covariates for matching. The matching results indicated that the propensity score distribution is nearly normal (Figure 2A), suggesting that the PSM model effectively captures the distribution characteristics of the covariates. After matching, most covariates had SMD less than 0.1, indicating good balance between the treatment group (*n* = 40) and the control group (*n* = 40) (Figures 2B,C). The QQ plot further validated that there were no significant differences in standard deviations before and after matching (*p* = 0.82), confirming the robustness of the matching process (Figure 2D). Subsequently, the univariate analysis revealed that the improvement in aphasia (*p* = 0.04) and the duration of treatment (*p* = 0.03) were significantly shortened in the Tongue Acupuncture treatment group (Supplementary Table S3). After Benjamini-Hochberg correction, the variables with false discovery rate (FDR)-adjusted *p* < 0.05 are Age, TSR, and improvement in aphasia.

**Figure 3:**
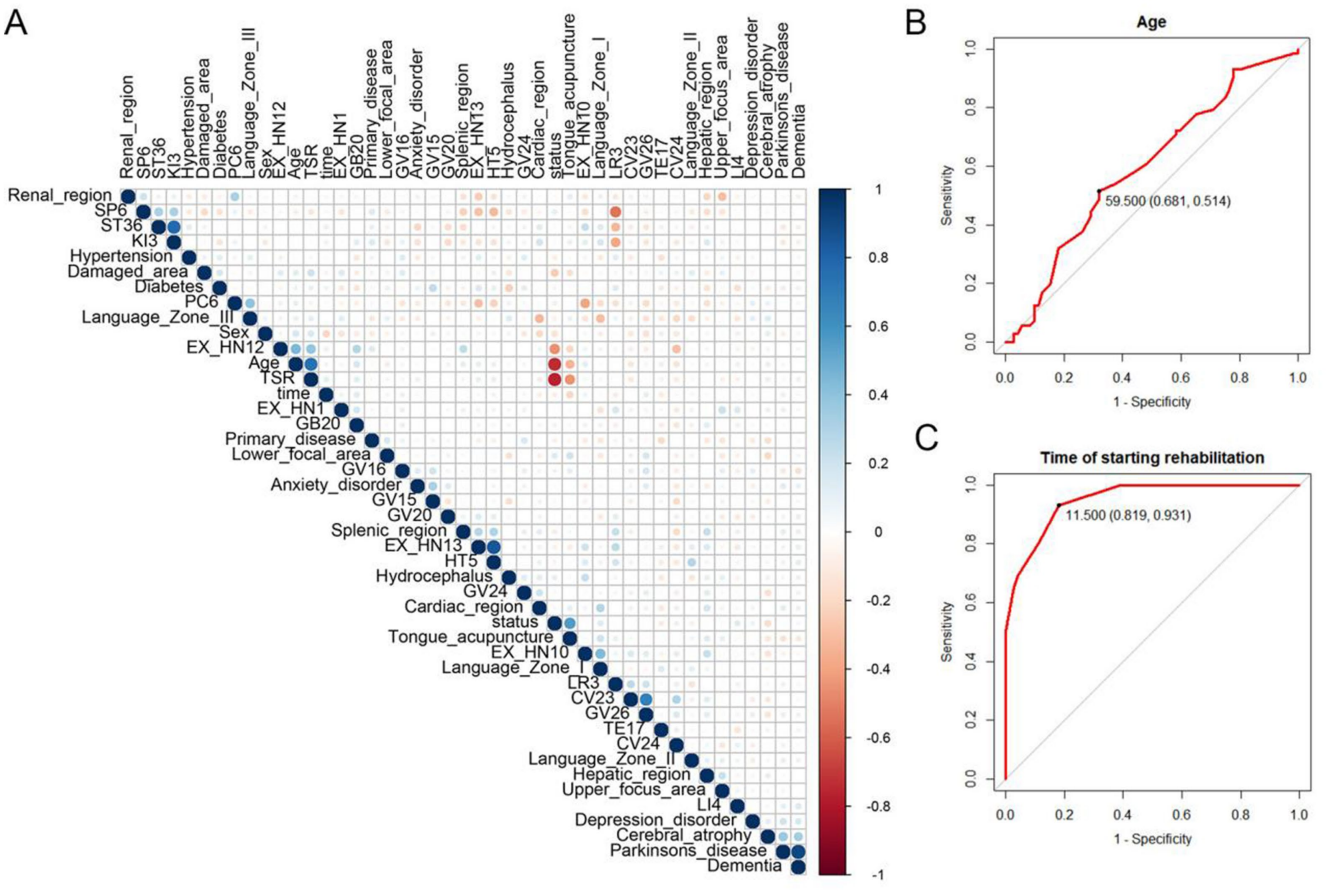
Data characteristics of patients with motor aphasia. **(A)** Correlation analysis of all clinical data of patients with motor aphasia. **(B,C)** The ROC curves show the optimal threshold points for age and the time to start rehabilitation as outcome variables for patients with significant improvement. TSR: time of starting rehabilitation.

We tested the correlation between all clinical data, and Pearson correlation analysis revealed that age, time to start of rehabilitation (TSR), EX-HN12, and brain injury area were significantly negatively correlated with the patient’s degree of improvement, while tongue-acupuncture treatment was significantly positively correlated with the patient’s degree of improvement (Figure 3A). Subsequently, we used the receiver operating characteristic curve to analyze the continuous variables to obtain the optimal threshold point. The optimal threshold for age was 65 years, and the optimal threshold for the time to start rehabilitation was 12 days (Figures 3B,C). Chi-square analysis was performed again, and there were statistically significant differences in age (chi-square test = 4.078, *p* = 0.043) and time to start rehabilitation (chi-square test = 10.267, *p* = 0.001) between the two groups of patients. Since we have a large number of potential variables and a relatively small number of cases, we will use machine learning algorithms to further screen the variables most closely related to the prognosis of motor aphasia.

### 3.2 Random forest analysis

**Figure 4:**
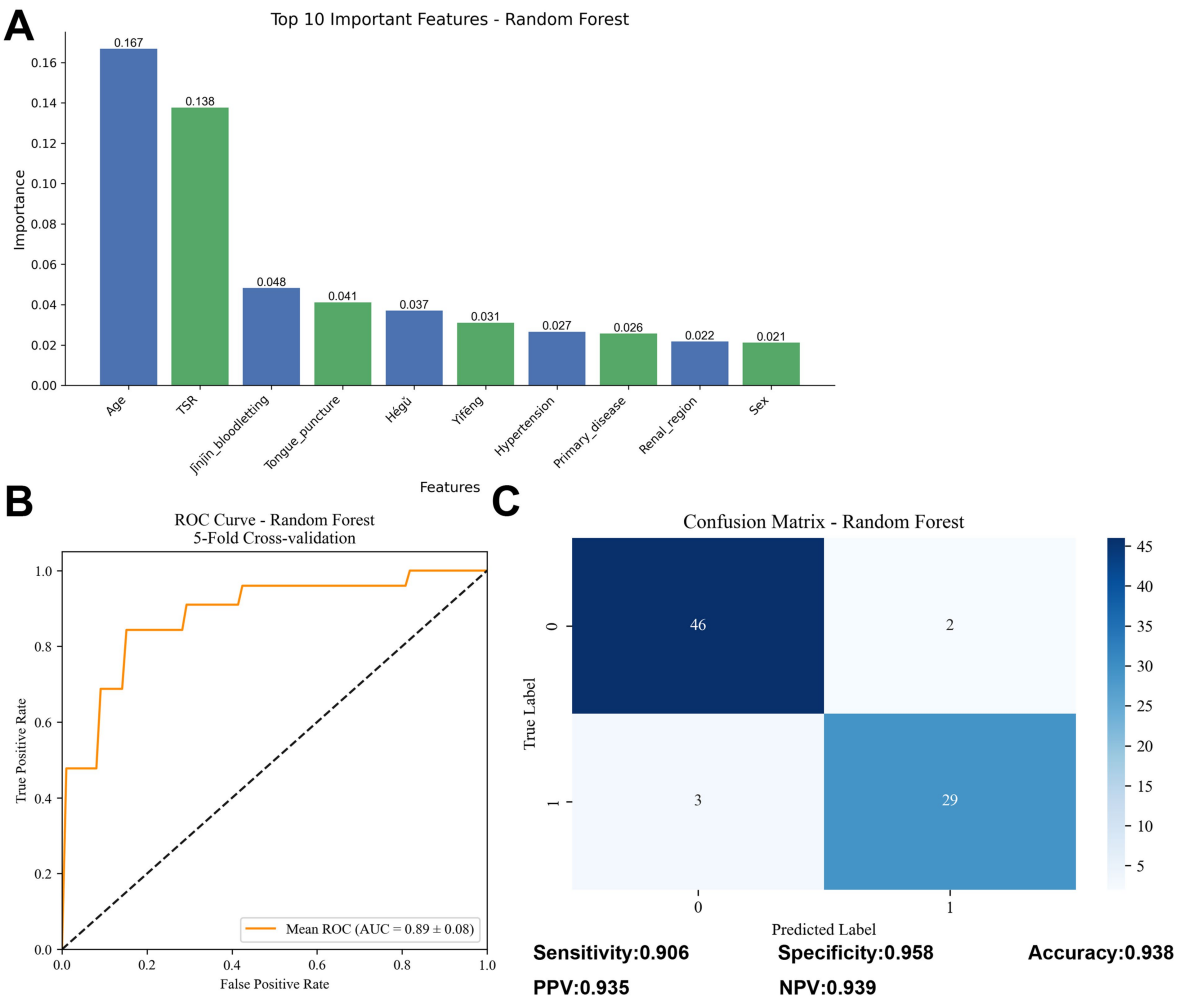
Results of Random Forest model analysis. **(A)** Bar chart of variable importance, displaying the top 10 variables impacting treatment outcomes for motor aphasia (based on Gini index reduction). **(B)** ROC curve, showing an average AUC of 0.89 ± 0.08 from five-fold cross-validation. **(C)** Confusion matrix, presenting the model’s sensitivity (90.6%), specificity (95.8%), and overall accuracy (93.8%). TSR, time of starting rehabilitation.

To assess the factors influencing treatment outcomes in patients with motor aphasia, we constructed a random forest model comprising 44 variables and 500 decision trees. The variables included demographic characteristics, clinical indicators, treatment methods, and other relevant factors. The random forest model evaluated the contribution of each variable to treatment outcomes by calculating their relative importance based on Gini index reduction. The top five influential variables identified were age (0.167), TSR(0.138), bloodletting at Jīnjīn point (0.048), tongue acupuncture (0.041), and Hégǔ (0.037) (Figure 4A).

To validate the model’s generalization capability, we conducted five-fold cross-validation, yielding an average AUC of 0.89 ± 0.08 (Figure 4B), indicating good predictive ability in distinguishing between effective and ineffective treatment cases. Confusion matrix analysis further revealed a sensitivity of 90.6%, specificity of 95.8%, and overall accuracy of 93.8% (Figure 4C). These metrics suggest that the model performed well on both training and validation data, demonstrating high predictive reliability and stability.

### 3.3 Lasso regression analysis

**Figure 5:**
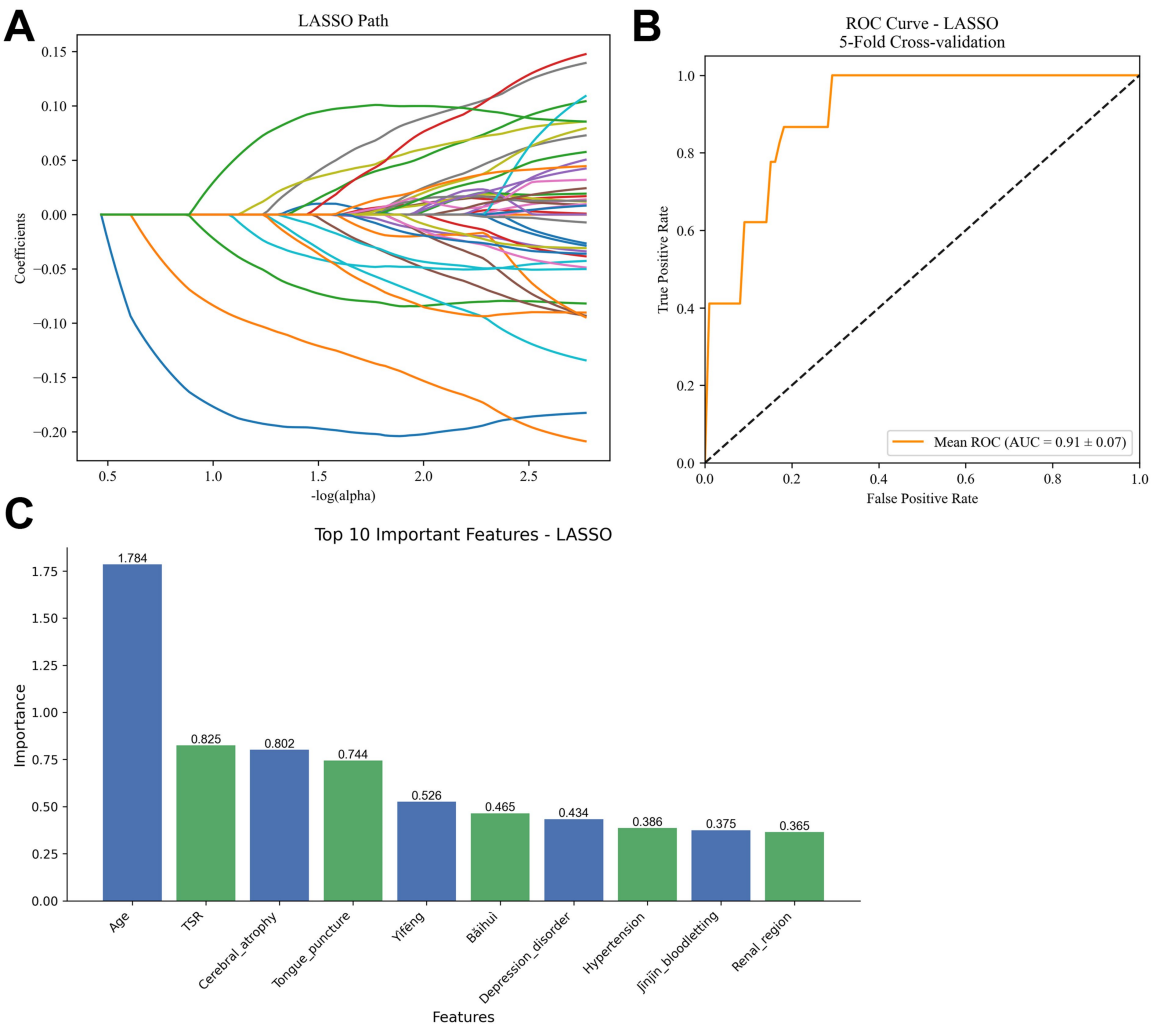
Results of lasso regression model analysis. **(A)** Coefficient path plot, illustrating the process of variable coefficients being compressed to zero as the penalty term lambda (*λ*) increases. **(B)** ROC curve, showing an average AUC of 0.91 ± 0.07 from five-fold cross-validation. **(C)** Bar chart of non-zero coefficients, presenting the magnitude of the top 10 variables (age, TSR, cerebral atrophy, tongue acupuncture, Yìfēng, etc.). TSR, time of starting rehabilitation.

To further optimize variable selection and develop a predictive model for treatment outcomes in motor aphasia, we employed Lasso regression, utilizing 10-fold cross-validation to select the penalty term lambda (*λ*) to minimize prediction error and achieve variable sparsity. As lambda increases, the model’s parameter estimation is progressively compressed; when lambda reaches a certain threshold, the coefficients of less important variables are reduced to zero, effectively excluding them from the model (Figure 5A). This process reduces model complexity while retaining the variables most relevant to treatment outcomes.

Model performance was evaluated using five-fold cross-validation, yielding an average AUC of 0.91 ± 0.07 (Figure 5B), indicating strong predictive ability in distinguishing effective from ineffective treatment cases. Confusion matrix analysis further revealed a sensitivity of 92.3%, specificity of 94.7%, and overall accuracy of 93.1%.

In terms of variable selection, Lasso regression identified several non-zero coefficient variables, ranked by the magnitude of their absolute values. The top five variables were: age (1.784), time to rehabilitation (TSR) (0.825), cerebral atrophy (0.802), tongue acupuncture (0.744), and Yìfēng (0.526). These coefficients reflect their relative contributions to predicting treatment outcomes (Figure 5C).

### 3.4 GBM algorithm analysis

To further assess the predictive model for treatment outcomes in motor aphasia, we developed a classification model based on Gradient Boosting Machine (GBM), utilizing a Bernoulli distribution to accommodate the binary classification problem. The model comprised 44 variables, constructed with 1,000 decision trees, a learning rate of 0.01, and a maximum tree depth of five to balance model complexity and predictive power.

**Figure 6:**
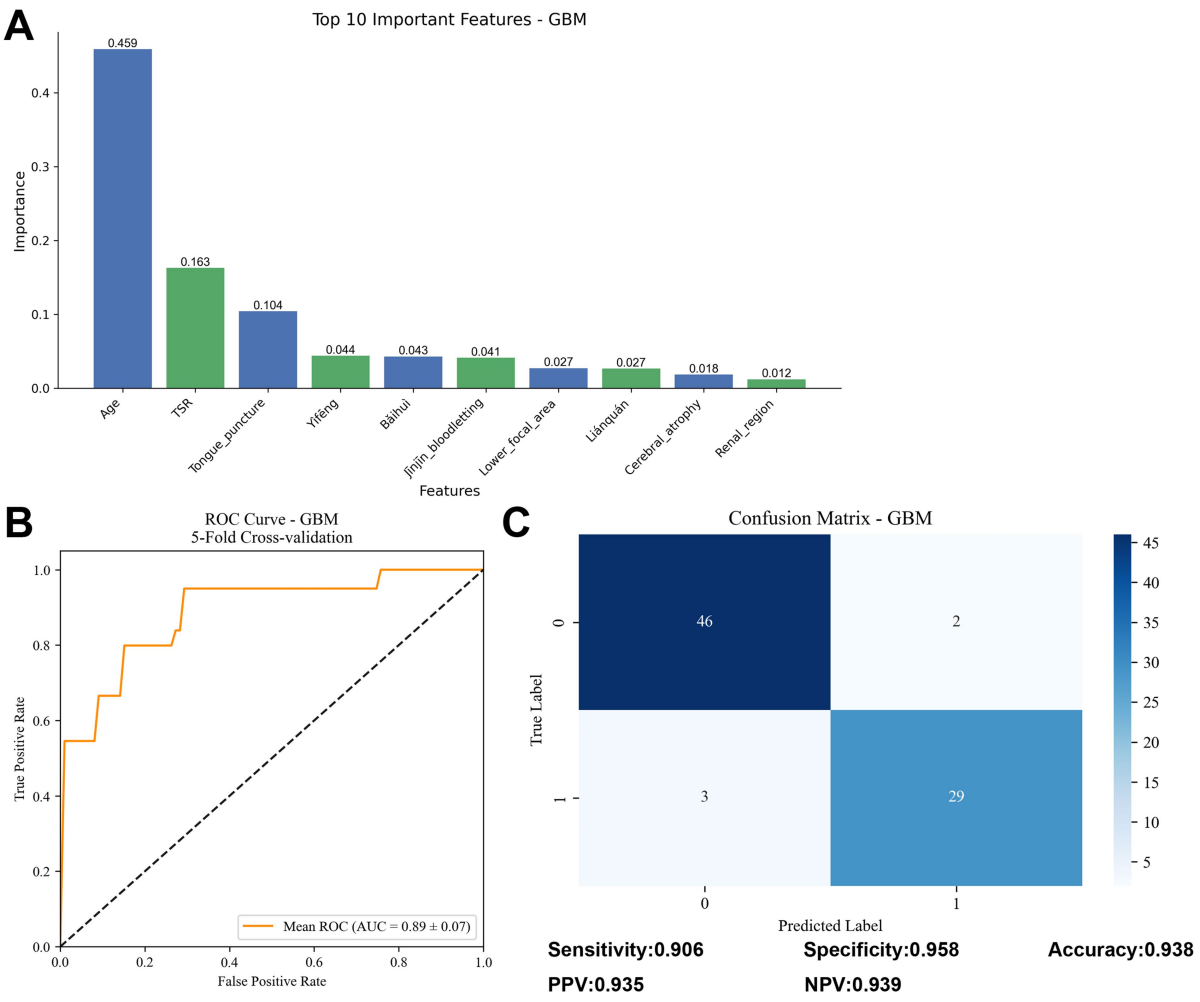
Results of GBM model analysis. **(A)** Bar chart of feature importance, displaying the ranking of the top 10 variables (age, TSR, tongue acupuncture, Yìfēng, Bǎihuì, etc.) based on gain. **(B)** ROC curve, showing an average AUC of 0.89 ± 0.07 from five-fold cross-validation. **(C)** Confusion matrix, presenting sensitivity (89.7%), specificity (90.6%), and accuracy (93.8%) on the test set.

Feature importance was analyzed based on gain, with the top five variables ranked as follows: age (0.459), time to rehabilitation (TSR) (0.163), tongue acupuncture (0.104), Yìfēng (0.044), and Bǎihuì (0.043) (Figure 6A). Model performance was evaluated using five-fold cross-validation, revealing an average AUC of 0.89 ± 0.07 (Figure 6B), indicating good predictive ability in distinguishing effective from ineffective treatment cases. On the test set, the GBM model achieved a sensitivity of 89.7%, specificity of 90.6%, and an overall accuracy of 93.8% (Figure 6C).

### 3.5 Cox survival analysis

Based on the variable importance analysis from GBM, Random Forest, and Lasso regression models, we identified age, TSR, and tongue acupuncture as key predictors of treatment outcomes in motor aphasia. To further evaluate the predictive value of these variables, we conducted univariate and multivariate Cox regression analyses.

**Figure 7:**
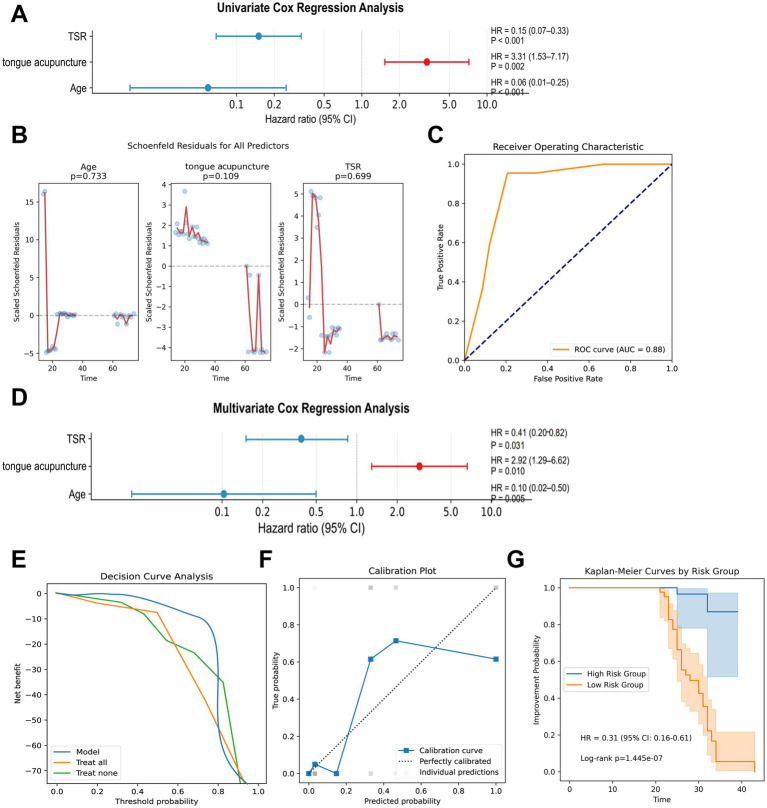
Results of cox regression model analysis. **(A)** Univariate Cox regression forest plot, displaying HR and 95% CI for age, TSR, and tongue acupuncture. **(B)** Schoenfeld residual plot, validating the proportional hazards assumption (*p* > 0.05). **(C)** ROC curve, indicating an AUC of 0.88 for the multivariate Cox regression model. **(D)** Multivariate Cox regression forest plot, presenting HR and 95% CI for age ≥ 60 years, TSR ≥ 12 points, and tongue acupuncture. **(E)** Decision curve analysis, showing net benefits of tongue acupuncture treatment within the threshold range of 0.2–0.8. **(F)** Calibration slope plot, illustrating the calibration of predicted probabilities against actual outcomes. **(G)** Kaplan–Meier curve, comparing treatment outcomes between low-risk and high-risk groups (HR = 0.31, *p* < 0.001).

Univariate Cox regression revealed that TSR (HR = 0.15, 95% CI 0.07–0.33, *p* < 0.001), age (HR = 0.06, 95% CI 0.01–0.25, *p* < 0.001), and tongue acupuncture (HR = 3.31, 95% CI 1.53–7.71, *p* = 0.002) were significantly associated with treatment outcomes in patients with motor aphasia (Figure 7A). We subsequently included these three variables in the multivariate Cox regression model. Schoenfeld residuals test indicated that the *p*-values for age (*p* = 0.773), TSR (*p* = 0.699), tongue acupuncture (*p* = 0.109), and the global model (*p* = 0.109) were all greater than 0.05, confirming the applicability of the proportional hazards (PH) assumption (Figure 7B) and supporting the reliability of the Cox regression model in this study.

The multivariate Cox regression model yielded an AUC of 0.88 (Figure 7C). The results indicated that age ≥ 60 years (HR = 0.10, 95% CI: 0.02–0.50, *p* = 0.005) is a risk factor for improved treatment outcomes, suggesting poorer prognosis for elderly patients; TSR ≥ 12 days (HR = 0.41, 95% CI: 0.20–0.82, *p* = 0.031) indicated lower improvement likelihood for patients starting tongue acupuncture treatment later; and tongue acupuncture treatment (HR = 2.92, 95% CI 1.29–6.62, *p* = 0.010) significantly enhanced treatment outcomes (Figure 7D). Multivariate logistic regression similarly indicates the impact of age, tongue acupuncture, and TSR on outcomes (Supplementary Figure S2). Decision curve analysis demonstrated significant net benefits for patients receiving tongue acupuncture within a threshold range of 0.2–0.8, indicating its clinical value (Figure 7E). The calibration slope plot showed predicted probabilities clustered around the diagonal, indicating good model calibration (Figure 7F).

To further validate the model’s robustness, we performed five-fold cross-validation and 1,000 bootstrap internal validations. The cross-validation C-index was 0.831 ± 0.06, with a Brier score of 0.194 ± 0.103 and a calibration slope of 0.817 ± 0.306; the bootstrap validation C-index was 0.802 ± 0.012, with a Brier score of 0.177 ± 0.025 and a calibration slope of 0.866 ± 0.18, all indicating strong predictive ability and calibration performance. Based on the multivariate Cox regression results, we categorized patients into low-risk (age <60 years, TSR < 12 points, receiving tongue acupuncture) and high-risk groups (age ≥60 years, TSR ≥ 12 points, not receiving tongue acupuncture). Cox regression analysis between the two groups showed significant differences (HR = 0.31, 95% CI 0.16–0.61, *p* < 0.001) (Figure 7G). These findings indicate that age, TSR, and tongue acupuncture are key factors in predicting treatment outcomes for motor aphasia, and that tongue acupuncture can significantly improve patient prognosis, making it particularly recommended for younger patients with better baseline language function.

## 4 Discussion

This study systematically analyzed predictive factors for treatment outcomes in patients with non-fluent aphasia using various machine learning algorithms, including LASSO regression, random survival forests, and gradient boosting machines, along with a multivariate Cox proportional hazards regression model. The findings revealed that tongue acupuncture was associated with significantly better treatment outcomes (HR = 2.92, 95% CI 1.29–6.62, *p* = 0.010), while age ≥60 years (HR = 0.10, 95% CI: 0.02–0.50, *p* = 0.005) and a rehabilitation initiation time of ≥12 days (HR = 0.41, 95% CI: 0.20–0.82, *p* = 0.031) were unfavorable factors affecting recovery. These findings provide essential insights for optimizing acupuncture treatment in non-fluent aphasia and emphasize the importance of early intervention and individualized patient factors.

Tongue acupuncture emerged as a key finding in this study, with its efficacy confirmed through a systematic analysis of 44 clinical variables, including various acupuncture points. Our machine learning approach controlled for minor variable influences, highlighting the independent effectiveness of tongue acupuncture. This finding aligns with previous studies demonstrating that tongue acupuncture significantly enhances language functions, such as repetition and naming abilities (31, 32). Unlike prior research that often combined tongue acupuncture with other points (33–35), this study distinctly identified the independent role of tongue acupuncture, offering a basis for standardizing clinical acupuncture protocols. This could not only simplify treatment processes and reduce patient burdens but also enhance therapeutic outcomes.

The study also found that age ≥60 years and rehabilitation commencement ≥12 days were associated with poorer recovery outcomes. Older patients may recover more slowly due to reduced neural plasticity, consistent with earlier research on language function recovery. Additionally, delayed rehabilitation initiation (TSR ≥ 12 days) correlated with worse prognosis, underscoring the importance of starting tongue acupuncture treatment early after the onset of non-fluent aphasia. Clinically, this suggests that tongue acupuncture should be integrated into early rehabilitation plans, potentially serving as a complementary method to speech and language therapy to optimize patient recovery.

This study evaluated key variables influencing the treatment outcomes of motor aphasia using LASSO regression, RSF, GBM, and Cox regression models. LASSO regression identified age (coefficient: 1.784), TSR (0.825), and tongue acupuncture (0.744) as high-impact variables (coefficients >0.5, range 0.5–2.0). The RSF model indicated age (importance: 0.167), TSR (0.138), bloodletting at Jingjin point (0.048), tongue acupuncture (0.041), and Hegu point (0.037) as significant variables (importance >0.03, range 0.03–0.2). The GBM model further validated the importance of age (gain: 0.459), TSR (0.163), and tongue acupuncture (0.104) (gain >0.1, range 0.1–0.5). Cox regression confirmed age ≥60 years (HR = 0.10), TSR ≥ 12 days (HR = 0.41), and tongue acupuncture (HR = 2.92) as significant predictors (HR < 0.5 or >2). These results indicate that age, TSR, and tongue acupuncture are consistently key factors affecting treatment outcomes across all models. The high importance of tongue acupuncture (LASSO coefficient: 0.744, RSF importance: 0.041, GBM gain: 0.104, HR: 2.92) supports its potential as an effective treatment for motor aphasia, consistent with previous studies on the positive impact of acupuncture on neurological recovery. The significant relevance of age and TSR underscores the need for clinical attention to elderly patients and the timing of early interventions. These findings provide data support for optimizing treatment protocols and directions for future research.

Although the specific mechanisms of tongue acupuncture require further investigation, existing evidence suggests that acupuncture stimulation at tongue points may activate language-related brain regions (e.g., left inferior temporal gyrus and Broca’s area) through neural network feedback (12, 31). These mechanisms provide possible explanations for the clinical findings of this study, but we emphasize the practical effects of tongue acupuncture in clinical practice rather than speculative mechanisms.

The strengths of this study include the use of multiple machine learning algorithms (LASSO, RSF, and GBM) to select key variables, further validated through the Cox regression model. By intersecting important variables from the three algorithms (age, TSR, and tongue acupuncture), we ensured the robustness of variable selection. Moreover, the multivariate Cox model confirmed the applicability of the proportional hazards assumption through Schoenfeld residual tests (*p* > 0.05), supporting the reliability of high and low-risk group stratification (HR = 0.31, 95% CI: 0.16–0.61, *p* < 0.001). This study validated the PH assumption, indicating the model’s reliability for predictions at different time points, providing significant evidence for clinical risk stratification. In the future, we will explore the practical applications of the model’s predictions, such as integrating them into clinical decision support systems to help doctors develop more precise treatment plans, enhancing the practicality of clinical practice.

However, this study has limitations. First, it is confined to a single-center design, with a small sample size and limited heterogeneity among patients, affecting the generalizability of the results. Secondly, we encountered constraints when analyzing the interactions between acupuncture and rehabilitation treatments, particularly in exploring the synergistic effects of tongue acupuncture and speech therapy. In the future, we plan to conduct multi-center studies to investigate potential synergistic effects on neurobiological mechanisms and clinical efficacy, providing a basis for optimizing combined treatment plans. Furthermore, while this study found independent efficacy for tongue acupuncture, the effects of other points (e.g., scalp points) remain to be validated. We intend to conduct randomized controlled trials to systematically evaluate the independent and synergistic effects of different acupuncture points, with the selection of relevant points based on existing literature and traditional medical theories. Thirdly, the implementation of Diagnosis-Intervention Packet (DIP) policy in this study standardized treatment courses, limiting in-depth analysis of dose–response relationships. Additionally, the lack of external validation necessitates further confirmation of the model and results in larger independent datasets. To address this, we plan to collaborate with multiple hospitals to obtain external data for validation, and explore data sharing mechanisms and unified statistical analysis methods to ensure the scientific integrity and consistency of the validation process. Future research integrating advanced neuroimaging techniques will further explore the long-term effects and mechanisms of tongue acupuncture, deepening the understanding of our findings.

## 5 Conclusion

This study confirms the significant effectiveness of tongue acupuncture in treating non-fluent aphasia and highlights the importance of early treatment and younger age in optimizing prognosis. These findings provide important references for standardizing clinical acupuncture treatments. Future multi-center, large-sample studies will further validate and expand these results, offering more evidence for the comprehensive management of non-fluent aphasia.

## Data Availability

The raw data supporting the conclusions of this article will be made available by the authors, without undue reservation.

## Glossary

ABC: Aphasia Battery of Chinese
AIC: Akaike Information Criterion
BIC: Bayesian Information Criterion
CILT: Constraint-Induced Language Therapy
CI: Confidence Interval
COX: Cox Proportional Hazards Model
GBM: Gradient Boosting Machine
HR: Hazard Ratio
KS: Kolmogorov–Smirnov
LASSO: Least Absolute Shrinkage and Selection Operator
MSE: Mean Squared Error
OOB: Out-of-Bag
OR: Odds Ratio
PH: Proportional Hazards
PSM: Propensity Score Matching
ROC: Receiver Operating Characteristic
RSF: Random Survival Forest
SLT: Speech and Language Therapy
SMD: Standardized Mean Difference
TSR: Time to Start Rehabilitation
TUF: Basic Form Processing
VIF: Variance Inflation Factor
WAB: Western Aphasia Battery
DIP: Diagnosis-Intervention Packet
STRICTA: Standards for Reporting Interventions in Clinical Trials of Acupuncture
FDR: False Discovery Rate
GCS: Glasgow Coma Scale
