# Supplementary material for: Machine learning predicts significant improvement in motor aphasia with tongue acupuncture
Source: Front Neurol. 2025 Oct 1;16:1554208. doi: 10.3389/fneur.2025.1554208 (PMC12520874; doi:10.3389/fneur.2025.1554208)
Supplement: Supplementary file 4 [file Data_Sheet_1.docx]

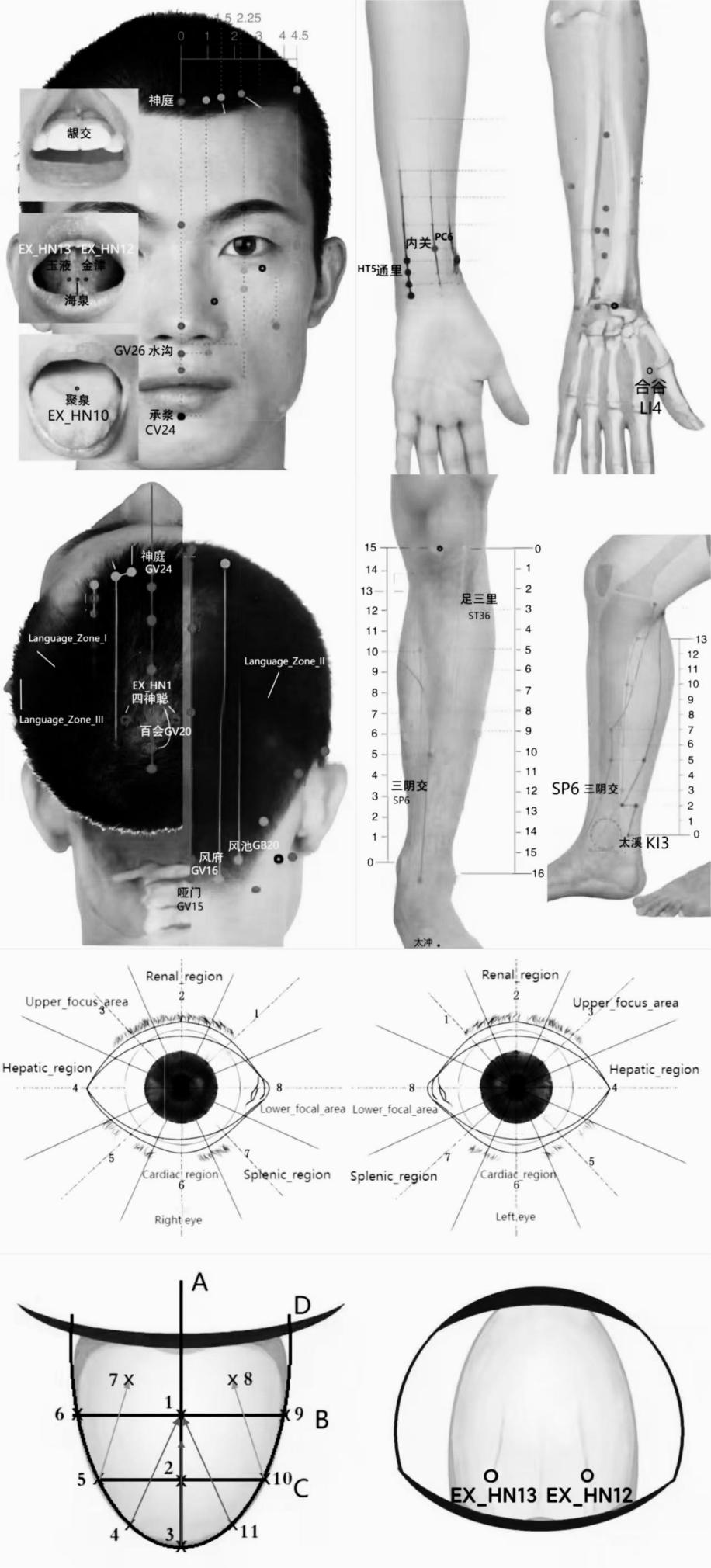


**Figure S1.Anatomical position of acupuncture and moxibustion points.The main acupuncture points involved in tongue acupuncture treatment include tongue acupuncture points and related head and neck meridian points, such as Lianquan (CV23), Jinjun (EX-HN12), Yuye (EX-HN13), and Chengjiang. These points are located in the oral and head-neck regions and are used to treat motor aphasia. The points shown in this illustration do not include ocular or retinal areas and are all situated in the head, face, or tongue, adhering to standard clinical practices.**
