# Supplementary figures and images for: Machine learning predicts significant improvement in motor aphasia with tongue acupuncture
Source: Front Neurol. 2025 Oct 1;16:1554208. doi: 10.3389/fneur.2025.1554208 (PMC12520874; doi:10.3389/fneur.2025.1554208)

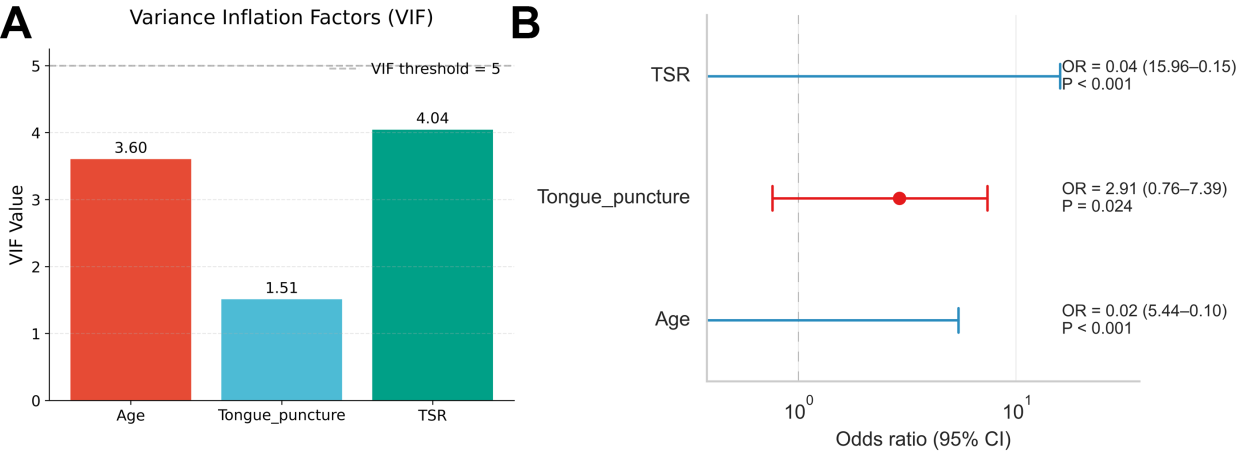


**Figure S2.Results of multivariate logistic regression.**

Supplement: Supplementary file 5 [file Data_Sheet_2.docx]
